# Reirradiation to the abdomen for gastrointestinal malignancies

**DOI:** 10.1186/1748-717X-4-55

**Published:** 2009-11-18

**Authors:** Waqar Haque, Christopher H Crane, Sunil Krishnan, Marc E Delclos, Milind Javle, Christopher R Garrett, Robert A Wolff, Prajnan Das

**Affiliations:** 1 Department of Radiation Oncology, The University of Texas M D Anderson Cancer Center, 1515 Holcombe Blvd, Houston, TX 77030, USA; 2 Department of Gastrointestinal Medical Oncology, The University of Texas M D Anderson Cancer Center, 1515 Holcombe Blvd, Houston, TX 77030, USA

## Abstract

**Background:**

Reirradiation to the abdomen could potentially play a role in palliation of symptoms or local control in patients with gastrointestinal malignancies. Our goal was to retrospectively determine rates of toxicity, freedom from local progression and overall survival in gastrointestinal cancer patients treated with reirradiation to the abdomen.

**Methods:**

Between November 2002 and September 2008, 13 patients with a prior history of abdominal radiotherapy (median dose 45 Gy) were treated with reirradiation for recurrent or metastatic gastrointestinal malignancies. The median interval between the two courses of radiotherapy was 26 months. Patients were treated with a hyperfractionated accelerated regimen, using 1.5 Gy fractions twice daily, with a median dose of 30 Gy (range 24-48 Gy). Concurrent chemotherapy was administered to 8 (62%) patients.

**Results:**

The 1-year rate of freedom from local progression was 50%, and the median duration of freedom from local progression was 14 months. The 1-year rate of overall survival was 62%, and the median duration of overall survival was 14 months. One patient developed grade 3 acute toxicity (abdominal pain and gastrointestinal bleeding), requiring hospitalization during radiotherapy; subsequently, that patient experienced a grade 4 late toxicity (gastrointestinal bleeding). No other patients developed grade 3-4 acute or late toxicity or required hospitalization during radiotherapy.

**Conclusion:**

Hyperfractionated accelerated reirradiation to the abdomen was well-tolerated with low rates of acute and late toxicity. Reirradiation could play a role in providing a limited duration of local control in gastrointestinal cancer patients with a history of prior abdominal radiotherapy.

## Introduction

Multiple studies have demonstrated the safety and efficacy of reirradiation at various sites of the body, including head and neck, brain, breast, lung and pelvis [1-15]. Among gastrointestinal malignancies, many studies have shown the safety and efficacy of pelvic reirradiation for rectal cancer [11-15]. However, to the best of our knowledge, no studies to date have evaluated the safety and efficacy of reirradiation to the abdomen for gastrointestinal malignancies. Reirradiation to the abdomen could potentially play a role in palliation of symptoms or local control. Hence, the goal of this study was to retrospectively determine rates of toxicity, freedom from local progression and overall survival in gastrointestinal cancer patients treated with reirradiation to the abdomen.

## Materials and methods

Between November 2002 and September 2008, 13 patients with gastrointestinal cancer and a history of prior abdominal radiotherapy underwent reirradiation, with a hyperfractionated accelerated approach, at the University of Texas M.D. Anderson Cancer Center. The hospital and radiotherapy records of these patients were reviewed. The M. D. Anderson Institutional Review Board approved this study.

### Patient Characteristics

Patient characteristics are shown in Table 1. The median age at the time of retreatment was 56 years (range 37-80 years). The diagnosis was pancreatic adenocarcinoma in 3 patients, colon adenocarcinoma colon in 3 patients, cholangiocarcinoma in 2 patients, and ampullary adenocarcinoma, gastric adenocarcinoma, duodenal adenocarcinoma, small bowel adenocarcinoma, and pancreatic neuroendocrine carcinoma, in 1 patient each.

**Table 1:** Patient and Treatment Characteristics

Characteristic	Median (Range) or Number of Patients (%)
Age at Retreatment (years)	56 (37-80)

Gender	
Male	9 (69%)
Female	4 (31%)

Pathology	
Pancreatic adenocarcinoma	3 (23%)
Colon carcinoma	3 (23%)
Cholangiocarcinoma	2 (15%)
Others*	5 (38%)

Prior Radiotherapy Dose	
30 Gy (10-12 fractions)	2 (15%)
35 Gy (14 fractions)	2 (15%)
45 Gy (25 fractions)	4 (31%)
50.4 Gy (28 fractions)	5 (38%)

Retreatment Interval (months)	26 (5-83)

Retreatment Dose**	
24 Gy	1 (8%)
30 Gy	6 (46%)
34.5 Gy	1 (8%)
39 Gy	4 (31%)
48 Gy	1 (8%)

Concurrent Chemotherapy	
Yes	8 (62%)
No	5 (38%)

Indication for Retreatment	
Palliation of Pain	5 (38%)
Palliation of Bleeding	4 (31%)
Definitive (Not candidate for other treatments)	3 (23%)
Consolidation after Chemotherapy	1 (8%)

* Ampullary, gastric, duodenal, small bowel, and pancreatic neuroendocrine

** Retreatment was given in 1.5 Gy twice daily fractions

Prior radiotherapy records were obtained and reviewed in all cases at the time of retreatment. The median dose of prior radiotherapy was 45 Gy (range 30-50.4 Gy). The prior radiotherapy dose was 30 Gy in 2.5-3 Gy fractions in 2 patients, 35 Gy in 2.5 Gy fractions in 2 patients, 45 Gy in 1.8 Gy fractions in 4 patients and 50.4 Gy in 1.8 Gy fractions in 5 patients. Prior radiation was given with a definitive intent in 9 patients and for palliation in 4 patients. The median interval between the two courses of radiotherapy was 26 months (range 5-83 months).

At the time of reirradiation, 8 (62%) patients had recurrent disease and 5 (38%) patients had metastatic disease. Prior to reirradiation, patients had received a median of 2 (range 0-4) different regimens of chemotherapy, not including concurrent chemotherapy given with radiation. Reirradiation was administered for palliation of pain in 5 patients, palliation of bleeding in 4 patients, definitive treatment in 3 patients who were not candidates for other therapies, and consolidative treatment after chemotherapy in 1 patient.

### Treatment

All patients underwent computed tomography (CT) simulation. Patient-specific information about reirradiation is shown in Table 2. Patients were treated with 150 cGy fractions twice daily, with an interval ≥ 6 hrs between fractions. The prescribed dose of reirradiation was 30 Gy in 7 patients, 39 Gy in 5 patients and 48 Gy in 1 patient. The fractionation regimen of 150 cGy twice daily and doses of 30-39 Gy were selected based on a similar regimen used for pelvic reirradiation at our institution [16]. The specific dose of 30 or 39 Gy was chosen by the attending radiation oncologist, based on the interval from the previous course of radiation and dose to critical structures. A higher dose of 48 Gy was selected in one patient because of limited overlap with prior fields and limited dose to critical structures. Radiation therapy was stopped early in 2 patients because of acute toxicity. Hence, the administered reirradiation dose was 24 Gy in 1 patient, 30 Gy in 6 patients, 34.5 Gy in 1 patient, 39 Gy in 4 patients and 48 Gy in 1 patient. The median administered dose of reirradiation was 30 Gy. In all cases, patients were reirradiated for recurrence or metastasis from the same primary tumor for which they were initially treated. The site of retreatment was para-aortic/paracaval nodes in 4 patients, pancreas in 3 patients, stomach in 2 patients, and the superior mesenteric region, duodenum, liver metastasis and abdominal wall mass, in 1 patient each. Among the 13 patients, the reirradiated region was completely within the previously treated volume in 9 patients, and the reirradiated region partially overlapped with the previously treated volume in 4 patients. Radiation therapy was delivered using intensity-modulated radiation therapy (IMRT) in 5 patients, 4-field conformal technique in 4 patients, wedge-pair in 2 patients, 5-field conformal technique in 1 patient, and two oblique fields in 1 patient. Reirradiation was administered to the gross tumor volume (GTV) with a 2-3 cm block margin for conformal plans. A margin of 1.5-2 cm was added to the GTV to form the planning target volume (PTV) for IMRT plans. Radiation therapy was delivered using 6-18 MV photons. In selected cases (N = 7), cumulative dose-volume histograms were obtained for the two courses of radiation therapy, with particular attention given to the cumulative doses to the spinal cord, kidneys, liver and bowel. Typical cumulative dose constraints included maximum dose to the spinal cord = 46 Gy, V_20 _for at least one kidney <33% and V_30 _for liver < 50%, although these could be exceeded at the discretion of the attending radiation oncologist, especially if there was a prolonged interval between the two courses of radiation therapy. Concurrent chemotherapy was administered to 8 (62%) patients, of whom 7 received capecitabine, and 1 received gemcitabine and erlotinib. None of the patients underwent surgical resection of the irradiated area.

**Table 2:** Patient-specific Retreatment Characteristics

**Patient No**.	Retreatment Site	Tumor Size (cm)*	Retreatment Dose (Gy)	Retreatment Interval (months)	Retreatment DVH	Cumulative DVH
1	Duodenum	7 × 7 × 5	30	42	Kidneys V_20 _0%, 38%, Liver V_30 _0%, Max. cord dose 15 Gy	

2	Stomach	10 × 4 × 2	30	29	Kidneys V_20 _0%, 0%, Liver V_30 _5%, Max. cord dose 20 Gy	

3	Liver	5 × 5 × 6	48	45	Kidneys V_20 _0%, 0%, Liver V_30 _25%, Max. cord dose 3 Gy	Kidneys V_20 _0%, 0%, Liver V_30 _58%, Max. cord dose 42 Gy

4	Stomach	10 × 9 × 10	30	5	Kidneys V_20 _0%, 2%, Max. cord dose 12 Gy	

5	Superior mesenteric	5 × 3 × 3	39	25	Kidneys V_20 _0%, 0%, Liver V_30 _0%, Max. cord dose 32 Gy	

6	Abdominal wall	7 × 3 × 10	30	26	Max. cord dose 12 Gy	

7	Para-aortic/caval	4 × 3 × 3	39	28	Kidneys V_20 _7%, 21%, Max. cord dose 23 Gy	Kidneys V_20 _22%, 32%, Max. cord dose 39 Gy

8	Pancreas	8 × 6 × 5	34.5	22	Kidneys V_20 _7%, 17%, Liver V_30 _5%, Max. cord dose 27 Gy	Kidneys V_20 _9%, 23%, Liver V_30 _22%, Max. cord dose 43 Gy

9	Pancreas	6 × 6 × 6	39	83	Kidney V_20 _0%, Liver V_30 _0%, Max. cord dose 12 Gy	Kidney V_20 _0%, Liver V_30 _12%, Max. cord dose 39 Gy

10	Para-aortic/caval	9 × 10 × 17	30	36	Max. cord dose 7 Gy	

11	Para-aortic/caval	9 × 6 × 8	30	26	Kidneys V_20 _0%, 0%, Liver V_30 _20%, Max. cord dose 18 Gy	Kidneys V_20 _14%, Liver V_30 _72%, Max. cord dose 46 Gy

12	Pancreas	6 × 4 × 6	24	12	Kidneys V_20 _0%, 0%, Liver V_30 _0%, Max. cord dose 17 Gy	Kidneys V_20 _8%, 24%, Liver V_30 _33%, Max. cord dose 46 Gy

13	Para-aortic/caval	5 × 6 × 5	39	11	Kidneys V_20 _5%, 8%, Liver V_30 _4%, Max. cord dose 23 Gy	Kidneys V_20 _18%, 20%, Liver V_30 _42%, Max. cord dose 38 Gy

DVH: Dose volume histogram

Max.: Maximum

* Maximum lateral × anterioposterior × craniocaudal dimensions, respectively

### Follow-up

Follow-up information was obtained from hospital records and radiation therapy department records. Follow-up information was also obtained from the M. D. Anderson Tumor Registry, which collects information on patients annually through letters, phone calls, and Bureau of Vital Statistics records. The median follow-up interval was 8 (range 3-26) months.

### Statistical Analysis

Acute and late toxicity was graded using the Common Terminology Criteria for Adverse Events version 3.0 [17]. Local progression was defined as any radiographic progression within the treated field. The rates of freedom from local progression and overall survival were estimated by Kaplan-Meier methods [18]. All time intervals were calculated from the date of completion of reirradiation.

## Results

### Local Control and Survival

**Figure 1:**
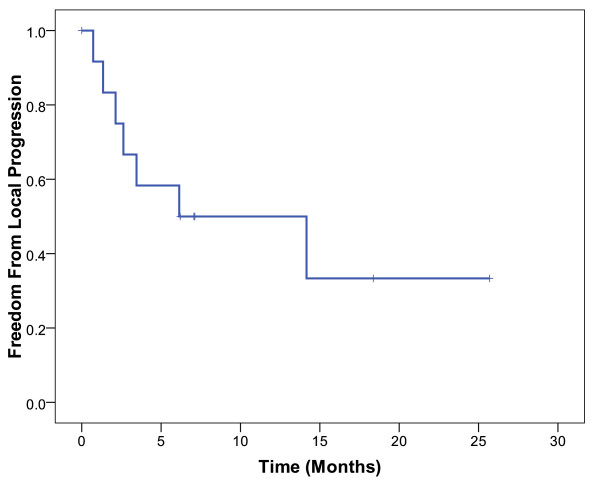
**Kaplan-Meier estimates of freedom from local progression in patients treated with abdominal reirradiation**.

**Figure 2:**
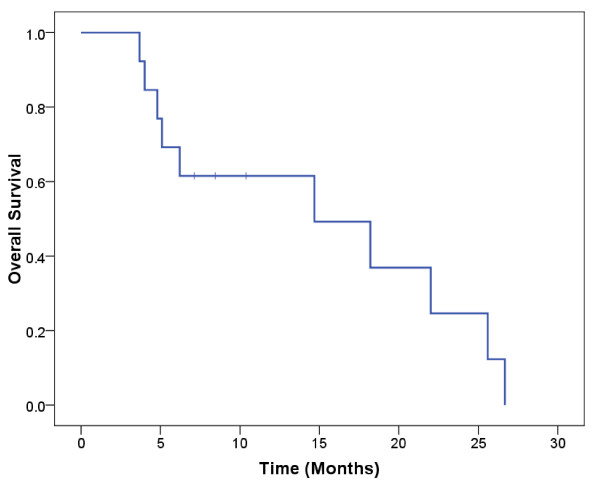
**Kaplan-Meier estimates of overall survival in patients treated with abdominal reirradiation**.

Seven (54%) of the 13 patients developed local progression. The 1-year actuarial rate of freedom from local progression was 50% (Figure 1). The median duration of freedom from local progression was 14 months. There were 10 deaths (77%) among the 13 patients. The 1-year actuarial overall survival rate was 62% (Figure 2). The median duration of overall survival was 14 months.

### Toxicity

Only 1 patient (8%) developed grade 3 acute toxicity (abdominal pain and bleeding from gastrojejunal anastomosis) during chemoradiation; this patient required hospitalization for 4 days and termination of radiation therapy before completion of the prescribed course. No other patient developed grade 3-4 acute toxicity or was hospitalized for acute toxicity. Only one patient developed grade 2 acute toxicity (duodenal ulceration and stricture); radiation therapy was stopped early in this patient to prevent progression of the ulcer. The remaining 11 patients completed their prescribed course of radiation therapy.

The patient with grade 3 acute toxicity subsequently developed grade 4 late toxicity (bleeding from the gastrojejunal anastomosis), two months after completion of reirradiation. The gastrojejunal anastomosis had received a cumulative maximum dose of 63 Gy in this patient. The patient was initially treated with endoscopic clips, epinephrine and coagulation, and then required a surgical revision of gastrojejunostomy. This patient had no subsequent episodes of gastrointestinal bleeding. No other patient developed grade 3-4 late toxicity.

## Discussion

We have hereby reported the first study on reirradiation to the abdomen for gastrointestinal cancers. Patients treated with reirradiation had a limited overall survival, with a median survival of 14 months, which reflects the poor prognosis of patients with recurrent or metastatic abdominal malignancies. Reirradiation provided local control for a limited duration, with a median duration of freedom from local progression of 14 months. However, this duration of local control was clinically significant, taking into consideration the limited life expectancy in these patients. Reirradiation provided these patients durable local control that lasted for the majority of their remaining life without significant increase in morbidity.

Reirradiation was well-tolerated with only one patient experiencing grade 3-4 acute and late toxicity. In addition, one patient developed grade 2 acute toxicity. These toxicity events involved gastrointestinal bleeding or ulceration. Patients treated with reirradiation may have a higher risk of developing gastrointestinal bleeding and ulceration, compared to patients not exposed to prior radiation therapy. Hence, we need to be cognizant about the risk of bleeding and ulceration in patients treated with abdominal reirradiation, especially around the duodenum or jejunal anastomosis.

We used a hyperfractionated accelerated regimen to deliver radiation therapy, with 150 cGy fractions, given twice daily. We recently reported outcomes in 50 rectal cancer patients treated with pelvic reirradiation with an identical regimen, with a total dose of 30-39 Gy [16]. Pelvic reirradiation was well-tolerated, with grade 3 acute toxicity occurring in 4% and grade 3-4 late toxicity occurring in 26% of patients. The median duration of freedom from local progression was 21 months and the median overall survival was 26 months. Rectal cancer patients likely had superior outcomes because of more favorable tumor biology compared to abdominal cancers, and because many of the rectal cancer patients underwent surgical resection in addition to radiation therapy. Nevertheless, we have now demonstrated in two separate sites of the body that reirradiation can be safely administered using a hyperfractionated accelerated approach, with 150 cGy twice daily fractions.

This study had several limitations. The number of patients was small, and there was considerable heterogeneity in tumor type and site of reirradiation. Hence, it is difficult to draw firm conclusions regarding the local control and survival outcomes in this study. Moreover, since patients received systemic therapy in addition to radiation therapy, it is difficult to surmise how much radiation contributed to the overall outcomes. Toxicity rates were assessed based on a review of hospital and departmental records, and therefore, may have been underestimated. The median follow-up interval was relatively short, and any patients who achieve long-term survival after reirradiation could potentially have higher rates of late toxicity. In spite of these limitations, this study shows that abdominal reirradiation could be a potential treatment option in selected patients with gastrointestinal malignancies. Of note, only 13 patients were treated with this approach in a period of about 6 years at a large institution. Careful patient selection clearly plays an important role in determining who might benefit from this treatment.

In conclusion, our study showed that abdominal reirradiation was well-tolerated with low rates of acute and late toxicity. Abdominal reirradiation appeared to provide local control, albeit with a limited duration. We suggest that abdominal reirradiation could have many potential applications in selected patients with recurrent or metastatic gastrointestinal cancers. Reirradiation may help in palliation of symptoms, such as pain or bleeding. In patients with isolated areas of disease that are refractory to chemotherapy, reirradiation could help achieve local control. In patients that have a good response to chemotherapy, reirradiation could have a consolidative role. Further studies are warranted to evaluate the role of abdominal reirradiation in these settings. Further studies are also needed to confirm the safety of abdominal reirradiation.

## Competing interests

The authors declare that they have no competing interests.

## Authors' contributions

WH participated in data collection and helped to draft the manuscript. PD conceived of the study, performed data analysis and helped to draft the manuscript. CHC helped in the design and coordination of the study. CHC, SK, MED, MJ, CRG, and RAW helped in data collection and manuscript revision. All authors read and approved the final manuscript.

## References

[B1] Tanvetyanon T Padhya T McCaffrey J Zhu W Boulware D Deconti R Trotti A Prognostic factors for survival after salvage reirradiation of head and neck cancer J Clin Oncol 2009 27 1983 1991 10.1200/JCO.2008.20.0691 19289616

[B2] Sulman EP Schwartz DL Le TT Ang KK Morrison WH Rosenthal DI Ahamad A Kies M Glisson B Weber R Garden AS IMRT reirradiation of head and neck cancer-disease control and morbidity outcomes Int J Radiat Oncol Biol Phys 2009 73 399 409 18556144 10.1016/j.ijrobp.2008.04.021

[B3] Popovtzer A Gluck I Chepeha DB Teknos TN Moyer JS Prince ME Bradford CR Eisbruch A The Pattern of Failure After Reirradiation of Recurrent Squamous Cell Head and Neck Cancer: Implications for Defining the Targets Int J Radiat Oncol Biol Phys 2009 74 1342 1347 19135312 10.1016/j.ijrobp.2008.10.042 PMC2765822

[B4] Janot F de Raucourt D Benhamou E Ferron C Dolivet G Bensadoun RJ Hamoir M Gery B Julieron M Castaing M Bardet E Gregoire V Bourhis J Randomized trial of postoperative reirradiation combined with chemotherapy after salvage surgery compared with salvage surgery alone in head and neck carcinoma J Clin Oncol 2008 26 5518 5523 10.1200/JCO.2007.15.0102 18936479

[B5] Langer CJ Harris J Horwitz EM Nicolaou N Kies M Curran W Wong S Ang K Phase II study of low-dose paclitaxel and cisplatin in combination with split-course concomitant twice-daily reirradiation in recurrent squamous cell carcinoma of the head and neck: results of Radiation Therapy Oncology Group Protocol 9911 J Clin Oncol 2007 25 4800 4805 10.1200/JCO.2006.07.9194 17947728

[B6] Henke G Paulsen F Steinbach JP Ganswindt U Isijanov H Kortmann RD Bamberg M Belka C Hypofractionated reirradiation for recurrent malignant glioma Strahlenther Onkol 2009 185 113 119 10.1007/s00066-009-1969-9 19240998

[B7] Paulino AC Mai WY Chintagumpala M Taher A Teh BS Radiation-induced malignant gliomas: is there a role for reirradiation? Int J Radiat Oncol Biol Phys 2008 71 1381 1387 18262733 10.1016/j.ijrobp.2007.12.018

[B8] Fokas E Wacker U Gross MW Henzel M Encheva E Engenhart-Cabillic R Hypofractionated Stereotactic Reirradiation of Recurrent Glioblastomas: A Beneficial Treatment Option after High-Dose Radiotherapy? Strahlenther Onkol 2009 185 235 240 10.1007/s00066-009-1753-x 19370426

[B9] Wurschmidt F Dahle J Petersen C Wenzel C Kretschmer M Bastian C Reirradiation of recurrent breast cancer with and without concurrent chemotherapy Radiat Oncol 2008 3 28 10.1186/1748-717X-3-28 18801165 PMC2556652

[B10] Cetingoz R Arican-Alicikus Z Nur-Demiral A Durmak-Isman B Bakis-Altas B Kinay M Is re-irradiation effective in symptomatic local recurrence of non small cell lung cancer patients? A single institution experience and review of the literature J Buon 2009 14 33 40 19373944

[B11] Valentini V Morganti AG Gambacorta MA Mohiuddin M Doglietto GB Coco C De Paoli A Rossi C Di Russo A Valvo F Bolzicco G Dalla Palma M Preoperative hyperfractionated chemoradiation for locally recurrent rectal cancer in patients previously irradiated to the pelvis: A multicentric phase II study Int J Radiat Oncol Biol Phys 2006 64 1129 1139 16414206 10.1016/j.ijrobp.2005.09.017

[B12] Mohiuddin M Lingareddy V Rakinic J Marks G Reirradiation for rectal cancer and surgical resection after ultra high doses Int J Radiat Oncol Biol Phys 1993 27 1159 1163 8262842 10.1016/0360-3016(93)90538-7

[B13] Mohiuddin M Marks GM Lingareddy V Marks J Curative surgical resection following reirradiation for recurrent rectal cancer Int J Radiat Oncol Biol Phys 1997 39 643 649 9336144 10.1016/s0360-3016(97)00340-4

[B14] Mohiuddin M Marks G Marks J Long-term results of reirradiation for patients with recurrent rectal carcinoma Cancer 2002 95 1144 1150 10.1002/cncr.10799 12209702

[B15] Lingareddy V Ahmad NR Mohiuddin M Palliative reirradiation for recurrent rectal cancer Int J Radiat Oncol Biol Phys 1997 38 785 790 10.1016/S0360-3016(97)00058-8 9240647

[B16] Das P Delclos M Skibber J Rodriguez-Bigas M Feig B Chang G Eng C Bedi M Krishnan S CH C Hyperfractionated Accelerated Radiotherapy for Rectal Cancer in Patients with Prior Pelvic Irradiation Int J Radiat Oncol Biol Phys 2009 69 S277 S277 10.1016/j.ijrobp.2009.04.056 19695792

[B17] Cancer Therapy Evaluation Program: Common Terminology Criteria for Adverse Events, version 3.0. Department of Health and Human Services, National Institutes of Health, National Cancer Institute 2003

[B18] Kaplan E Meier P Nonparametric estimation from incomplete observations J Am Stat Assoc 1958 53 457 481 10.2307/2281868

